# Imaging Features by Machine Learning for Quantification of Optic Disc Changes and Impact on Choroidal Thickness in Young Myopic Patients

**DOI:** 10.3389/fmed.2021.657566

**Published:** 2021-04-29

**Authors:** Dandan Sun, Yuchen Du, Qiuying Chen, Luyao Ye, Huai Chen, Menghan Li, Jiangnan He, Jianfeng Zhu, Lisheng Wang, Ying Fan, Xun Xu

**Affiliations:** ^1^Department of Ophthalmology, Shanghai General Hospital, Shanghai Jiao Tong University School of Medicine, Shanghai, China; ^2^National Clinical Research Center for Eye Diseases, Shanghai, China; ^3^Shanghai Key Laboratory of Ocular Fundus Disease, Shanghai, China; ^4^Shanghai Engineering Center for Visual Science and Photo Medicine, Shanghai, China; ^5^Shanghai Engineering Center for Precise Diagnosis and Treatment of Eye Diseases, Shanghai, China; ^6^Department of Preventative Ophthalmology, Shanghai Eye Disease Prevention and Treatment Center, Shanghai Eye Hospital, Shanghai, China; ^7^Department of Automation, Institute of Image Processing and Pattern Recognition, Shanghai Jiao Tong University, Shanghai, China

**Keywords:** myopia, machine learning, radiomics, optic disc, choroidal thickness

## Abstract

**Purpose:** To construct quantifiable models of imaging features by machine learning describing early changes of optic disc and peripapillary region, and to explore their performance as early indicators for choroidal thickness (ChT) in young myopic patients.

**Methods:** Eight hundred and ninety six subjects were enrolled. Imaging features were extracted from fundus photographs. Macular ChT (mChT) and peripapillary ChT (pChT) were measured on swept-source optical coherence tomography scans. All participants were divided randomly into training (70%) and test (30%) sets. Imaging features correlated with ChT were selected by LASSO regression and combined into new indicators of optic disc (IODs) for mChT (IOD_mChT) and for pChT (IOD_pChT) by multivariate regression models in the training set. The performance of IODs was evaluated in the test set.

**Results:** A significant correlation between IOD_mChT and mChT (*r* = 0.650, *R*^2^ = 0.423, *P* < 0.001) was found in the test set. IOD_mChT was negatively associated with axial length (AL) (*r* = −0.562, *P* < 0.001) and peripapillary atrophy (PPA) area (*r* = −0.738, *P* < 0.001) and positively associated with ovality index (*r* = 0.503, *P* < 0.001) and torsion angle (*r* = 0.242, *P* < 0.001) in the test set. Every 1 × 10 μm decrease in IOD_mChT was associated with an 8.87 μm decrease in mChT. A significant correlation between IOD_pChT and pChT (*r* = 0.576, *R*^2^ = 0.331, *P* < 0.001) was found in the test set. IOD_pChT was negatively associated with AL (*r* = −0.478, *P* < 0.001) and PPA area (*r* = −0.651, *P* < 0.001) and positively associated with ovality index (*r* = 0.285, *P* < 0.001) and torsion angle (*r* = 0.180, *P* < 0.001) in the test set. Every 1 × 10 μm decrease in IOD_pChT was associated with a 9.64 μm decrease in pChT.

**Conclusions:** The study introduced a machine learning approach to acquire imaging information of early changes of optic disc and peripapillary region and constructed quantitative models significantly correlated with choroidal thickness. The objective models from fundus photographs represented a new approach that offset limitations of human annotation and could be applied in other areas of fundus diseases.

## Introduction

Myopia is one of the major causes of visual impairment, of which the prevalence has been increasing worldwide in recent decades (1–3). East Asia bears a high incidence of myopic maculopathy in absolute terms (4, 5). The dramatically increasing prevalence makes it urgent to commence early control of myopia in China (6).

The progression of myopia is accompanied by various characteristic changes of optic disc and peripapillary region. With the elongation of axial length (AL), optic disc tilt and torsion appear in the beginning due to the oblique orientation of the vertical axis (7–9). With further development, characteristic features in the temporal adjacent area appear, including peripapillary atrophy (PPA), a crescent-shaped atrophic chorioretinal abnormality, and increasing disc fovea distance (10–13). In addition, the onset of chorioretinal atrophy, which leads to a gradual decrease of choroidal thickness (ChT), has become a common concern in predicting the progression of high myopia and pathological myopia. Subfoveal ChT correlated positively with visual acuity and negatively with axial elongation in mild myopia in a cohort study aged from 14 to 65 years (14). In highly myopic eyes without macular pathology, mean macular ChT (mChT) and peripapillary ChT (pChT) are important predictive factors of visual acuity (15, 16). Moreover, subfoveal ChT is an independent predictor for myopic maculopathy progression in high myopes in a recent 2-year longitudinal study (17).

Therefore, investigation of the association between optic disc changes and ChT in myopia is necessary for mining imaging indicators of optic disc (IODs) for the progression of choroid thinning and pathological myopia. However, the subtle and complex changes of optic disc and peripapillary region in the early stage of myopia are not fully explained by a few manually measured features. It is urgent to find new objective and quantifiable methods for the thorough exploration of optic disc changes and their association with ChT.

Radiomics, as a new image processing method, aims to decompose and quantify characteristics of medical imaging and construct models for disease diagnosis based on engineered hard-coded algorithms (18, 19). For eye diseases, clinical diagnosis requires a variety of imaging examinations. Color fundus photographs provide abundant information on morphology, color and texture of fundus, which brings favorable advantages for the utility of radiomics. The imaging features of optic disc and peripapillary region in patients with myopia have not been studied to construct quantifiable models for the progression of choroid thinning and pathological myopia.

The present study decided optic disc and peripapillary region as the regions of interest (ROIs) and managed to acquire imaging features from fundus photographs for the construction of IODs, and further explored their performance as early indicators for changes of ChT in young myopic patients.

## Materials and Methods

### Setting and Participants

The cross-sectional study was authorized by the Ethics committee of Shanghai General Hospital, Shanghai Jiao Tong University, Shanghai, China and followed the tenets of the Declaration of Helsinki. The subjects in this study included students attending Shanghai University in October 2018. All subjects have understood the study protocol and signed informed consent forms. The protocol was consistent with those of previous studies (20, 21).

The systolic and diastolic blood pressures of all subjects were measured and calculated by the following formula to obtain the mean arterial pressure (MAP): (SBP + 2 × DBP)/3. All subjects received comprehensive ophthalmic examinations including refractive error assessment using an autorefractor instrument (model KR-8900; Topcon, Tokyo, Japan), measurement of best-corrected visual acuity (BCVA) and intraocular pressure (IOP) (Full Auto Tonometer TX-F; Topcon), slit-lamp biomicroscope, color-fundus examination, and measurement of the thickness of choroid, retina, and nerve fiber layer using swept-source optical coherence tomography (SS-OCT; model DRI OCT-1 Atlantis; Topcon). Anterior chamber depth (ACD), and AL were measured using optical low-coherence reflectometry (Lenstar; Haag-Streit AG, Koeniz, Switzerland). Subjective refraction was performed for all subjects by a trained optometrist. The spherical equivalent refraction (SER) was defined as the sphere plus half a cylinder. The BCVA was converted into the logarithm of minimal angle resolution (logMAR). The medical history of all subjects was recorded in detail. Each participant underwent all examinations on the same day.

All enrolled participants met the inclusion criteria as follows: age between 16 and 40 years; SER < 0.5 diopter (D); IOP 21 mm Hg or less; normal anterior chamber angles and normal depth of the anterior chamber; with a healthy optic nerve head without glaucomatous damage; and no peripapillary retinal nerve fiber layer thickness (pRNFLT) changes on both eyes. All subjects older than 40 years were excluded, as the incidence of glaucoma is associated with age, and the lenticular changes from aging might have an effect on the myopic refractive error. The other exclusion criteria were as follows: a history of ocular or major systemic diseases, including congenital cataract and glaucoma, hypertension, and diabetes; a history of previous intraocular or refractive surgery; a history of glaucoma among first-degree family members; and other evidence of retinal pathology. Generally, except for the optic disc and peripapillary changes associated with myopia, all participants had no other ocular abnormalities. Only the right eye of each subject was selected for statistical analysis.

### SS-OCT Imaging

All subjects underwent the examination of SS-OCT by an experienced examiner from 10:00 a.m. to 3:00 p.m. to minimize the influence of diurnal variation (22, 23). The SS-OCT machine of the present study was equipped with a light source of 1,050 μm wavelength and a scanning speed of 100,000 A-scans per second, with which a depth resolution of 8 μm and a lateral resolution of 10 μm of ocular tissue was reached. Before image taking by SS-OCT scanning, spherical power diopter, cylindrical power diopter and AL, with which the SS-OCT machine calculated the scan circle size, were input to minimize the error caused by the magnification factors associated with AL. The device operator adjusted the focus settings to the specific eye model of each participant before scanning. The scan protocol used the 12-line radial scan pattern with a resolution of 1,024 × 12 centered on the fovea and optic disc. All measurements in OCT images were acquired by a single experienced technician. The SS-OCT was performed twice for the first 30 participants to assess measurement reproducibility. Images with a signal strength index of 60 or less were excluded. The borderlines of layers were identified by the built-in software, which segmented each layer automatically. Manual adjustments were performed whenever inaccurate auto segmentation of each layer led to measurement artifacts.

The average thickness of choroid, retina, and nerve fiber layer was automatically calculated using built-in software. The topographic maps were overlapped to an Early Treatment Diabetic Retinopathy Study (ETDRS) grid (6 × 6 mm) that was focused on the macula or optic disc. In this way, each scan was divided into nine regions as follows: three concentric circles including the 1-mm diameter central circle, the 3-mm diameter inner circle, and the 6-mm diameter outer circle defined the inner ring (the area between the central circle and the inner circle) and the outer ring (the area between the inner circle and the outer circle), which were further divided into four quadrants—namely, temporal, superior, nasal, and inferior. ChT was measured as the vertical distance between the Brunch's membrane and the choroidal–scleral interface. Macular retinal thickness (mReT) was measured as the vertical distance between the internal limiting membrane (ILM) and the interface between the photoreceptor outer segments and retinal pigment epithelium. pRNFLT was measured as the vertical distance between the ILM and the interface between the nerve fiber layer and the ganglion cell layer. The average thickness of all nine sectors was calculated in the macular region. While in the peripapillary area, only four regions of the outer ring were applied because no choroidal tissue was found in the central sector and the inner ring, and the topographic maps were not reliable in these regions (20, 21).

### Extraction of Imaging Feature Pool on Color Fundus Photograph

**Figure 1:**
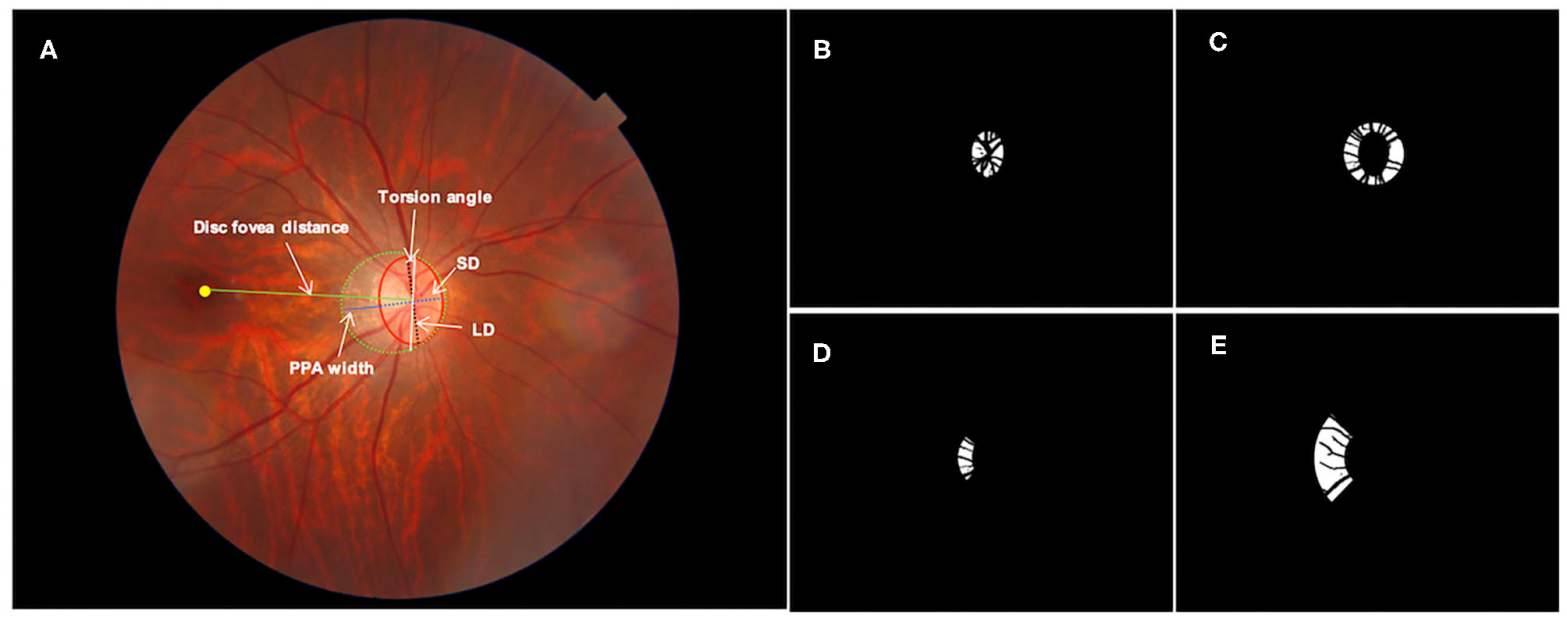
Definition of regions of interest for imaging feature extraction: **(A)** original fundus photograph. The optic disc (red solid line), peripapillary atrophy (PPA, green dotted line) and macular fovea (yellow dot) were annotated manually. The white arrows indicate PPA width (blue solid line), disc fovea distance (green solid line), the longest diameter (LD, black dotted line) and shortest diameter (SD, blue dotted line) of the optic disc. The ovality index was defined as the ratio between the LD and SD of the optic disc. Torsion angle was measured between the LD and the vertical line (white solid line) 90° from the line connecting the fovea and the center of the optic disc. **(B)** Mask of optic disc; **(C)** mask of the inner ring; **(D)** mask of the inner temporal region and **(E)** mask of the outer temporal region. Vessels were excluded for imaging feature extracting.

Red-free and color fundus photographs centered on the optic disc were imaged by the same SS-OCT machine with a digital, non-mydriatic retinal camera. ROIs for feature extraction were delineated as follows: the margins of optic disc and PPA were carefully marked with an in-house annotation software as shown in Figure 1A in random order and in a masked mode, not knowing the participants' background and medical history, as described previously (7, 20). The optic disc margin was defined as the inner border of the peripapillary scleral ring. The degree of optic disc tilt was measured by the ovality index, defined as the ratio of the shortest-longest disc diameters (Figure 1A) (24). Torsion angle referred to the angle between the line perpendicular to the line connecting macula and the center of optic disc and the long axis of optic disc. Inferotemporal and superonasal torsion correlated with negative and positive values (Figure 1A) (25). PPA margin was defined as an inner crescent of chorioretinal atrophy with good visibility of the large choroidal vessels and the sclera (13). The peripapillary region was divided into eight regions as follows: optic disc margin and two Early Treatment Diabetic Retinopathy Study (ETDRS) concentric circles including the 3-mm diameter inner circle, and the 6-mm diameter outer circle defined the inner ring (the area between the optic disc margin and the inner circle) and the outer ring (the area between the inner circle and the outer circle), which were further divided into four quadrants—temporal, superior, nasal, and inferior. The imaging features were extracted from four ROIs including the optic disc (Figure 1B), the inner ring (Figure 1C), the inner temporal region (Figure 1D) and the outer temporal region (Figure 1E), separately. Vessels were excluded for imaging feature extracting.

Radiomic features describing characteristics of morphology, color and texture were acquired with the PyRadiomics package (19) implemented under Python software (Python 3.7, Python Software Foundation, Beaverton). PyRadiomics operators were input into Python in advance. Operators employed in this study were as follows: operators for morphology including 10 2D shape features; operators for color including 19 first-order statistical features; operators for texture including 24 Gray Level Cooccurrence Matrix (GLCM) features, 16 Gray Level Run Length Matrix (GLRLM) features, 16 Gray Level Size Xone Matrix (GLSM) features, 5 Neighboring Gray Tone Difference Matrix (NGTDM) features and 14 Gray Level Dependence Matrix (GLDM) features. Original photographs were transferred into three separate channels of LAB color space prior to feature extraction as the PyRadiomics package only applied to single-channel images. Operators above extracted radiomic features of designated ROIs automatically after being provided with images and corresponding masks of ROIs. The total number of pixels was converted into square millimeters or millimeters and the magnification was corrected for AL by applying Littmann's formula (26). The package for operators above was detailed on the website (https://pyradiomics.readthedocs.io/en/latest/).

### Model Construction of Imaging Indicators

**Figure 2:**
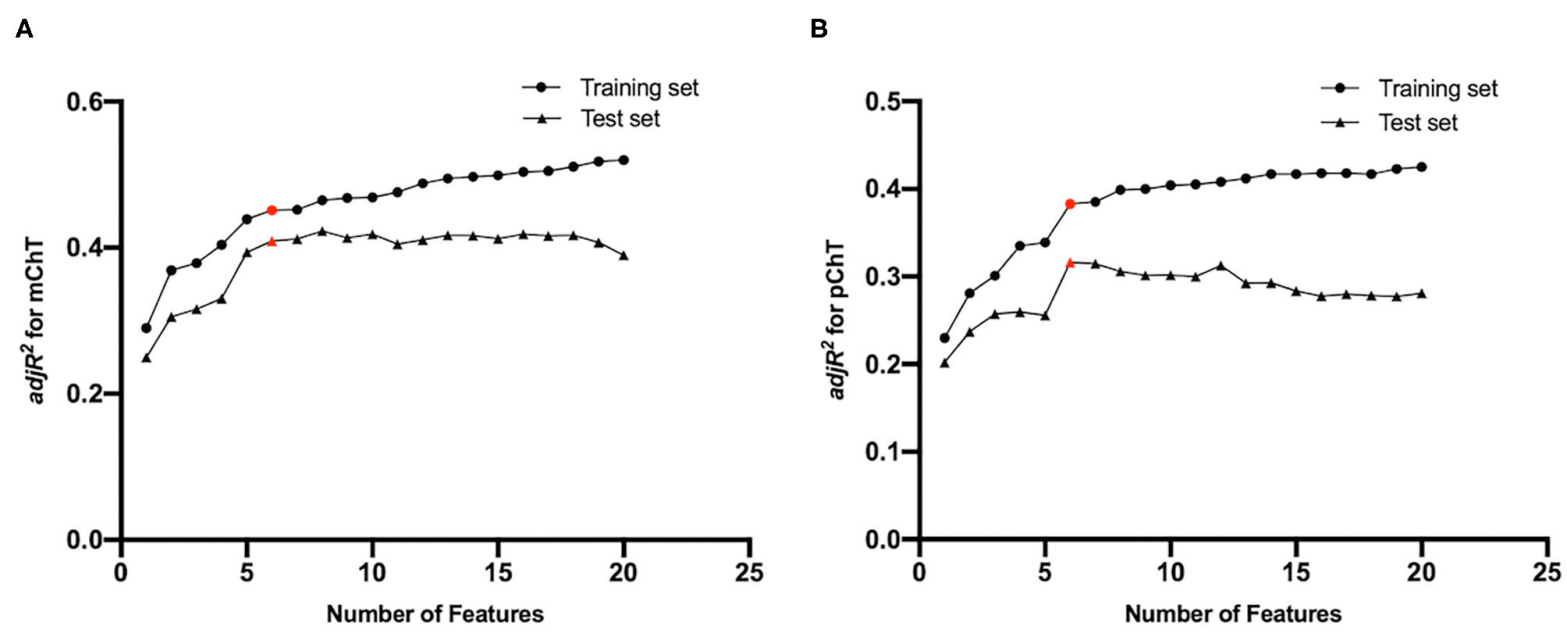
Feature selection results by LASSO regression for **(A)** macular choroidal thickness (mChT) and **(B)** peripapillary choroidal thickness (pChT). As the number of selected features increased, adjusted coefficients of determination (*adjR*^2^) in the training and test sets fluctuated. Red points represent selected feature sets.

All imaging features from fundus photographs were included in the final feature pool. All participants were divided into the training set (70%) and the test set (30%). LASSO regression was performed to determine the most relevant features with choroidal thickness and to exclude variables with multi-collinearity. Briefly, LASSO regression was performed in the training set. Selected features were used to constructed IODs for ChT with the multivariate regression model. Adjusted coefficients of determination (*adjR*^2^) were calculated in the training and test sets separately. The models of six features were selected because *adjR*^2^ was arising in both training and test sets until the number of selected features increased to six (Figure 2). With more features enrolled, *adjR*^2^ increased slowly in the training set and decreased in the test set because of redundancy and overfitting. The IOD for mChT (IOD_mChT) were constructed with PPA width, disc fovea distance, skewness of intensity in A channel of OT, range of intensity in L channel of IT, range of intensity in A channel of OT, range of intensity in B channel of OT. The IOD for pChT (IOD_pChT) were constructed with PPA perimeter, skewness of intensity in B channel of I, skewness of intensity in A channel of OT, range of intensity in L channel of IT, range of intensity in A channel of OT, range of intensity in B channel of OT. The definitions of imaging features were detailed on the website (https://pyradiomics.readthedocs.io/en/latest/).

### Statistical Analysis

SPSS software (IBM SPSS Statistics 21; SPSS, Inc, Chicago, IL) was used for all statistical analysis. All characteristics were shown in form as means ± standard deviation for continuous data and as counts for categorical data. The distribution of all variables was examined for normality using the Kolmogorov-Smirnov test. Student's *t*-test and the χ^2^ test were used to determine differences between the training and test sets.

The performance of IODs was evaluated by calculating Spearman's correlation with ChT and validated imaging features of the optic disc in the test sample. Partial correlation analysis adjusted for sex and age was performed to investigate the relationship between pChT or mChT and features of optic disc and peripapillary region. To assess the impact of optic disc changes on ChT, features with significant associations during the univariate analysis (*P* < 0.05) were included in the final multivariate regression analysis. In order to further explore the impact of IODs on ChT in people with different degrees of myopia, subjects were divided into three groups based on AL, as described previously (27, 28): AL < 24 mm, AL 24 mm to <26 mm, and AL 26 mm or more. The third group belonged to high myopia. Standardized regression coefficients and adjusted coefficients of determination (*adjR*^2^) were calculated in all subjects and each group separately.

Statistical significance was set as *P* < 0.05 (two-sided).

## Results

### General Characteristics

All participants were randomly divided into the training (70.0%) and test (30.0%) sets. The demographic and ocular characteristics of the training and test sets are shown in Table 1. The mean SER was −4.98 ± 3.10 D in the training set and −4.73 ± 3.05 D in the test set. The mean AL of the training and test sets were 25.52 ± 1.35 mm and 25.50 ± 1.32 mm. No significant difference was found in all ocular parameters between the training and test sets.

**Table 1:** Demographic and ocular characteristics of the training and test sets.

	**Total** **(*N* = 896)**	**Training set** **(N = 627)**	**Test set** **(*N* = 269)**	***P***
Age, y	20.65 ± 3.04	20.80 ± 3.15	20.29 ± 2.74	0.014^*^
Sex, male/female	395/501	285/342	110/159	0.207
MAP, mm Hg	89.11 ± 11.11	89.38 ± 11.19	88.49 ± 10.90	0.271
IOP, mm Hg	14.00 ± 2.83	13.98 ± 2.81	14.01 ± 2.89	0.884
ACD, mm	3.60 ± 0.34	3.59 ± 0.34	3.61 ± 0.32	0.385
SER, diopter	−4.91 ± 3.08	−4.98 ± 3.10	−4.73 ± 3.05	0.266
BCVA, logMAR	0.02 ± 0.06	0.02 ± 0.05	0.03 ± 0.07	0.447
AL, mm	25.51 ± 1.34	25.52 ± 1.35	25.50 ± 1.32	0.884
PPA area, mm^2^	0.68 ± 0.63	0.69 ± 0.61	0.67 ± 0.68	0.711
Ovality index	0.79 ± 0.10	0.78 ± 0.10	0.79 ± 0.09	0.226
Torsion angle, deg	4.98 ± 19.78	4.95 ± 19.87	5.06 ± 19.61	0.935
IOD_mChT, 10 μm	21.25 ± 4.21	21.19 ± 4.25	21.41 ± 4.12	0.475
IOD_pChT, 10 μm	14.34 ± 2.78	14.34 ± 2.84	14.34 ± 2.67	0.987
mChT, μm	211.00 ± 62.03	211.88 ± 63.02	208.94 ± 59.71	0.515
pChT, μm	143.33 ± 45.58	143.45 ± 45.46	143.05 ± 45.94	0.903
mReT, μm	276.01 ± 12.72	275.85 ± 13.14	276.40 ± 11.70	0.547
pRNFLT, μm	91.93 ± 10.04	91.68 ± 9.91	92.50 ± 10.33	0.268

*MAP, mean arterial pressure; IOP, intraocular pressure; ACD, anterior chamber depth; SER, spherical equivalent refraction; BCVA, best-corrected visual acuity; logMAR, logarithm of minimal angle resolution; AL, axial length; PPA, peripapillary atrophy; mChT, macular choroidal thickness; pChT, peripapillary choroidal thickness; IOD_mChT, the indicator of optic disc for mChT; IOD_pChT, the indicator of optic disc for pChT; mReT, macular retinal thickness; pRNFLT, peripapillary retinal nerve fiber layer thickness*.

* *Significant difference*.

### Performance of Imaging Indicators

**Figure 3:**
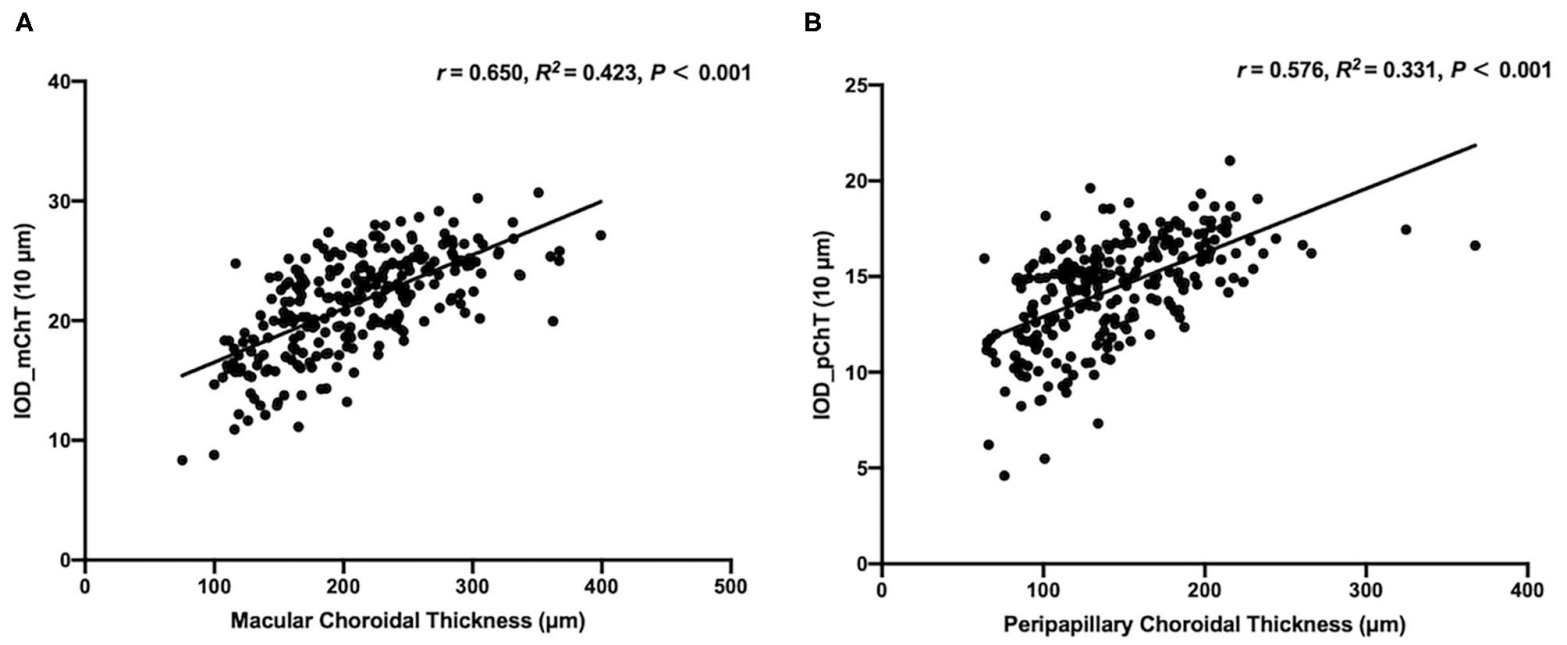
Linear correlation between the macular choroidal thickness (mChT) and the indicator of optic disc for mChT (IOD_mChT) in the test set **(A)** and linear correlation between the peripapillary choroidal thickness (pChT) and the indicator of optic disc for pChT (IOD_pChT) in the test set **(B)**.

To assess the performance of IODs, Spearman's correlation between IODs and clinical features associated with the progression of choroid thinning and pathological myopia was analyzed in the test set and shown in Table 2 and Linear correlation between IODs and ChT in the test set was shown in Figure 3. The mean IOD_mChT of the test set was (21.41 ± 4.12) × 10 μm, whereas the average mChT of the corresponding participants was 208.94 ± 59.71 μm. A significant correlation between IOD_mChT and mChT (*r* = 0.650, *R*^2^ = 0.423, *P* < 0.001) was found. Moreover, IOD_mChT was negatively associated with AL (*r* = −0.562, *P* < 0.001), and PPA area (*r* = −0.738, *P* < 0.001). IOD_mChT was positively associated with ovality index (*r* = 0.503, *P* < 0.001) and torsion angle (*r* = 0.242, *P* < 0.001). The mean IOD_pChT was (14.34 ± 2.67) × 10 μm and the mean average pChT was 143.05 ± 45.94 μm in the test set. There was also a very strong correlation between IOD_pChT and pChT (*r* = 0.576, *R*^2^ = 0.331, *P* < 0.001). IOD_pChT was negatively associated with AL (*r* = −0.478, *P* < 0.001) and PPA area (*r* = −0.651, *P* < 0.001). IOD_pChT was positively associated with ovality index (*r* = 0.285, *P* < 0.001) and torsion angle (*r* = 0.180, *P* = 0.003).

**Table 2:** Correlation analysis between constructed IODs with clinical features in the test set.

	**IOD_mChT, 10 μm**		**IOD_pChT, 10 μm**	
	***r***	***P***	***r***	***P***
mChT, μm	0.650	< 0.001^*^	0.641	< 0.001^*^
pChT, μm	0.490	< 0.001^*^	0.576	< 0.001^*^
AL, mm	−0.562	< 0.001^*^	−0.478	< 0.001^*^
PPA area, mm^2^	−0.738	< 0.001^*^	−0.651	< 0.001^*^
Ovality index	0.503	< 0.001^*^	0.285	< 0.001^*^
Torsion angle, deg	0.242	< 0.001^*^	0.180	0.003^*^

*mChT, macular choroidal thickness; pChT, peripapillary choroidal thickness; IOD_mChT, the indicator of optic disc for mChT; IOD_pChT, the indicator of optic disc for pChT; AL, axial length; PPA, peripapillary atrophy*.

* *Significant difference*.

**Figure 4:**
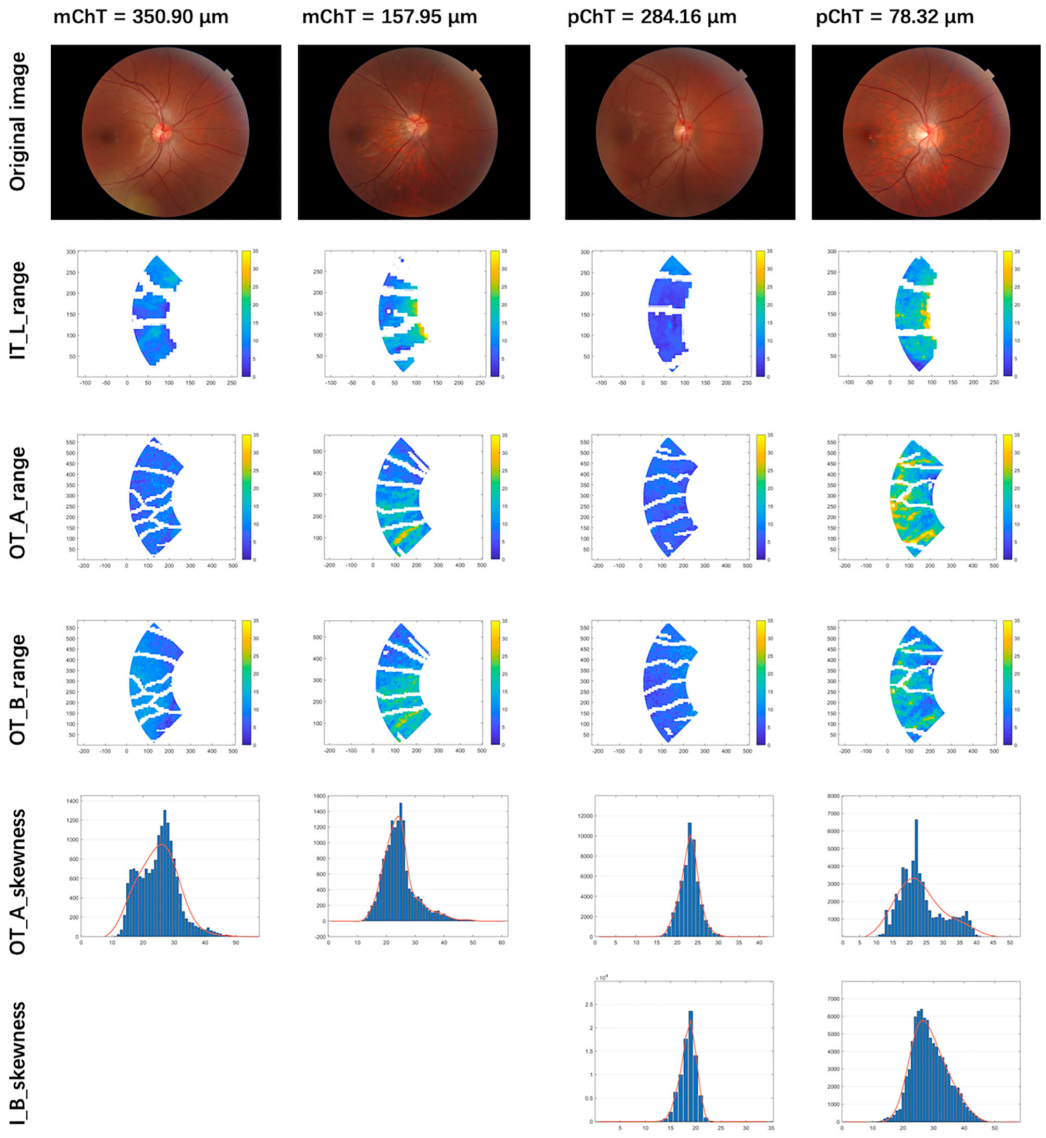
Visualization of selected radiomic features. Column 1 and 2: patients with macular choroidal thickness (mChT) of 350.90 and 157.95 μm. Column 3 and 4: patients with peripapillary choroidal thickness (pChT) of 284.16 and 78.32 μm. Row 1: original images. Row 2: range of intensity in L channel of the inner temporal region (IT_L_range); Row 3: range of intensity in A channel of the outer temporal region (OT_A_range); Row 4: range of intensity in B channel of the outer temporal region (OT_B_range). Row 5: skewness of intensity in A channel of the outer temporal region (OT_A_skewness); Row 6: skewness of intensity in B channel of the inner ring (I_B_skewness). Higher values of range mean larger variations of intensity in the LAB channel of color. Higher values of skewness mean larger proportions of the brighter region.

The visualization of enrolled radiomic features in the models of IODs was illustrated in Figure 4.

### Correlation Between mChT and Imaging Features of Optic Disc and Peripapillary Region

Partial correlation coefficient between mChT and features of optic disc and peripapillary region after adjusting for sex and age are summarized in Table 3. mChT was positively associated with IOD_mChT (*r* = 0.664, *P* < 0.001), followed by SER (*r* = 0.450, *P* < 0.001), ovality index (*r* = 0.332, *P* < 0.001), and torsion angle (*r* = 0.192, *P* < 0.001). mChT was negatively associated with AL (*r* = −0.497, *P* < 0.001), PPA area (*r* = −0.492, *P* < 0.001), BCVA (*r* = −0.121, *P* < 0.001).

**Table 3:** Partial correlation analysis between macular choroidal thickness and features of the optic disc adjusted for sex and age in all subjects.

	**Total (*****N*** **= 896)**	
	***r***	***P***
MAP, mm Hg	0.052	0.120
IOP, mm Hg	0.072	0.032^*^
ACD, mm	0.039	0.239
SER, diopter	0.450	< 0.001^*^
BCVA, logMAR	−0.121	< 0.001^*^
AL, mm	−0.497	< 0.001^*^
PPA area, mm^2^	−0.492	< 0.001^*^
Ovality index	0.332	< 0.001^*^
Torsion angle, deg	0.192	< 0.001^*^
IOD_mChT, 10 μm	0.664	< 0.001^*^

*MAP, mean arterial pressure; IOP, intraocular pressure; ACD, anterior chamber depth; SER, spherical equivalent refraction; BCVA, best-corrected visual acuity; logMAR, logarithm of minimal angle resolution; AL, axial length; PPA, peripapillary atrophy; IOD_mChT, the indicator of optic disc for macular choroidal thickness*.

* *Significant difference*.

Multivariate regression analysis adjusted for age and sex was constructed to identify independent factors associated with mChT as shown in Table 4. Because the current study focused on the features of optic disc and peripapillary region, and the explanatory power of the AL was greater than that of the spherical equivalent, only variations in AL and features of optic disc were used for multivariate regression analysis. The analysis showed that sex (*P* = 0.001), AL (*P* < 0.001) and IOD_mChT (*P* < 0.001) were independently associated with mChT in all subjects. According to the model, every 1 × 10 μm decrease in IOD_mChT was associated with an 8.87 μm decrease in mChT. After being stratified by myopia severity, 119 subjects belonged to the group with AL < 24 mm (13.3%), 464 subjects belonged to the group with AL 24 mm to <26 mm (51.8%), and 313 subjects belonged to the group with AL 26 mm or more (34.9%). The multivariate model showed that IOD_mChT correlated positively with mChT in all three groups (all, *P* < 0.001) while AL was only associated with mChT in the group with AL 24 mm to <26 mm (*P* = 0.039). The overall *adjR*^2^ in all subjects was 0.460 and the *adjR*^2^ was 0.319 for AL < 24 mm, 0.290 for AL 24 mm to <26 mm, and 0.447 for AL 26 mm or more.

**Table 4:** Multivariate regression analysis of association with macular choroidal thickness in all subjects and groups with different degrees of myopia.

	**Total** **(*****N*** **= 896)**			**AL < 24 mm** **(*****N*** **= 119)**			**24 mm ≤ AL < 26 mm** **(*****N*** **= 464)**			**AL ≥ 26 mm** **(*****N*** **= 313)**		
	***B***	**β**	***P***	***B***	**β**	***P***	***B***	**β**	***P***	***B***	**β**	***P***
Age, y	−0.20	−0.01	0.697	−0.13	−0.01	0.936	0.15	0.01	0.853	−1.05	−0.06	0.167
Sex, male/female	−10.74	−0.09	0.001^*^	−21.84	−0.18	0.036^*^	−11.36	−0.10	0.020^*^	−7.47	−0.07	0.111
AL, mm	−7.41	−0.16	< 0.001^*^	−19.07	−0.15	0.093	−9.54	−0.09	0.039^*^	0.65	0.01	0.839
PPA area, mm^2^	5.33	0.05	0.177	9.33	0.04	0.746	−0.12	0.00	0.987	6.59	0.10	0.149
Ovality index	−2.03	0.00	0.917	−54.34	−0.08	0.435	4.69	0.01	0.882	−14.03	−0.03	0.611
Torsion angle, deg	0.11	0.04	0.179	0.08	0.03	0.686	0.02	0.01	0.910	0.22	0.08	0.063
IOD_mChT, 10 μm	8.87	0.60	< 0.001^*^	11.61	0.57	< 0.001^*^	8.40	0.49	< 0.001^*^	8.88	0.71	< 0.001^*^

*AL, axial length; PPA, peripapillary atrophy; IOD_mChT, the indicator of optic disc for macular choroidal thickness. Adjusted for all variables listed. adjR^2^ for all subjects = 0.460; adjR^2^ for AL < 24 mm = 0.319; adjR^2^ for AL 24 mm to <26 mm = 0.290; adjR^2^ for AL 26 mm or more = 0.447*.

* *Significant difference*.

### Correlation Between pChT and Imaging Features of Optic Disc and Peripapillary Region

Partial correlation coefficient between pChT and morphological features of optic disc and peripapillary region after adjusting for sex and age are summarized in Table 5. pChT was positively associated with IOD_pChT (*r* = 0.604, *P* < 0.001), followed by SER (*r* = 0.363, *P* < 0.001), ovality index (*r* = 0.124, *P* < 0.001), torsion angle (*r* = 0.124, *P* < 0.001). pChT was negatively associated with AL (*r* = −0.406, *P* < 0.001), PPA area (*r* = −0.351, *P* < 0.001) and BCVA (*r* = −0.103, *P* = 0.002).

**Table 5:** Partial correlation analysis between peripapillary choroidal thickness and features of the optic disc adjusted for sex and age in all subjects.

	**Total (*****N*** **= 896)**	
	***r***	***P***
MAP, mm Hg	0.066	0.050
IOP, mm Hg	0.089	0.008^*^
ACD, mm	0.082	0.014^*^
SER, diopter	0.363	< 0.001^*^
BCVA, logMAR	−0.103	0.002^*^
AL, mm	−0.406	< 0.001^*^
PPA area, mm^2^	−0.351	< 0.001^*^
Ovality index	0.124	< 0.001^*^
Torsion angle, deg	0.124	< 0.001^*^
IOD_pChT, 10 μm	0.604	< 0.001^*^

*MAP, mean arterial pressure; IOP, intraocular pressure; ACD, anterior chamber depth; SER, spherical equivalent refraction; BCVA, best-corrected visual acuity; logMAR, logarithm of minimal angle resolution; AL, axial length; PPA, peripapillary atrophy; IOD_pChT, the indicator of optic disc for peripapillary choroidal thickness*.

* *Significant difference*.

Multivariate regression analysis adjusted for age and sex was constructed to identify independent factors associated with pChT as shown in Table 6. The analysis showed that sex (*P* = 0.015), AL (*P* < 0.001), PPA area (*P* = 0.044), IOD_pChT (*P* < 0.001) were independently associated with pChT. According to the model, every 1 × 10 μm decrease in IOD_pChT was associated with a 9.64 μm decrease in pChT. With stratification of myopia severity, the multivariate model showed that IOD_pChT correlated positively with pChT in all three groups (all, *P* < 0.001) while AL was only associated with pChT in the group with AL < 24 mm (*P* = 0.009). The overall *adjR*^2^ for pChT yielded 0.389 while the *adjR*^2^ were 0.367, 0.286, and 0.339 in the group with AL < 24 mm, 24 mm to <26 mm, and 26 mm or more, respectively.

**Table 6:** Multivariate regression analysis of association with peripapillary choroidal thickness in all subjects and groups with different degrees of myopia.

	**Total** **(*****N*** **= 896)**			**AL < 24 mm** **(*****N*** **= 119)**			**24 mm ≤ AL < 26 mm** **(*****N*** **= 464)**			**AL ≥ 26 mm** **(*****N*** **= 313)**		
	***B***	**β**	***P***	***B***	**β**	***P***	***B***	**β**	***P***	***B***	**β**	***P***
Age, y	−0.64	−0.04	0.113	−0.72	−0.05	0.558	−0.48	−0.03	0.420	−1.35	−0.10	0.036^*^
Sex, male/female	−6.17	−0.07	0.015^*^	−19.03	−0.20	0.016^*^	−5.17	−0.06	0.156	−5.12	−0.06	0.197
AL, mm	−5.30	−0.16	< 0.001^*^	−22.42	−0.22	0.009^*^	−5.38	−0.07	0.118	−1.39	−0.03	0.606
PPA area, mm^2^	5.90	0.08	0.044^*^	29.45	0.17	0.159	0.14	0.00	0.978	5.19	0.10	0.153
Ovality index	−29.44	−0.06	0.051	28.58	0.05	0.586	−36.93	−0.08	0.119	−43.97	−0.10	0.057
Torsion angle, deg	0.04	0.02	0.493	0.32	0.16	0.040^*^	−0.08	−0.04	0.411	0.07	0.04	0.461
IOD_pChT, 10 μm	9.64	0.59	< 0.001^*^	12.25	0.56	< 0.001^*^	9.87	0.54	< 0.001^*^	8.71	0.61	< 0.001^*^

*AL, axial length; PPA, peripapillary atrophy; IOD_pChT, the indicator of optic disc for peripapillary choroidal thickness. Adjusted for all variables listed. adjR^2^ for all subjects = 0.389; adjR^2^ for AL < 24 mm = 0.367; adjR^2^ for AL 24 mm to <26 mm = 0.286; adjR^2^ for AL 26 mm or more = 0.339*.

* *Significant difference*.

## Discussion

To the best of our knowledge, this is the first study to construct objective and quantifiable models of imaging indicators describing early changes of the optic disc and peripapillary region and further assess its impact on choroidal thickness. Our results have demonstrated a positive correlation between constructed IODs and ChT, even in the highly myopic group. According to features enrolled in IOD models which were visualized in Figure 4, thinner mChT and pChT were associated with a larger variation of color intensity and a larger proportion of brighter area in the peripapillary region, which represented changes of fundus tissue structure that could not be described objectively but only perceived subjectively without the method of radiomics and machine learning. Moreover, the models suggested thinner mChT was associated with larger PPA, longer disc fovea distance while thinner pChT was associated with larger PPA, all of which were consistent with those of previous studies (7, 11, 29–32).

In the early stage of myopia, the optic disc and peripapillary region are undergoing complex changes that can only be perceived through the ophthalmologists' senses but indescribable and unquantifiable. Several morphological changes of the optic disc and PPA have been reported to be significantly associated with the progression of high myopia and pathological myopia (7, 11, 20, 29–31). As discussed above, choroidal thinning, which appears before visible lesions of pathological myopia, has been widely discussed as a relatively reliable indicator for future progression of high myopia and pathological myopia (14–17). All these studies highlighted the importance of early changes of the optic disc and peripapillary region as a risk indicator for choroid thinning and future progression of pathological myopia.

However, the above indicators have several obvious shortcomings. First, these clinic features were measured manually and highly depends on the ophthalmologists' subjective estimation. When measured by different ophthalmologists, different results may occur due to the lack of uniform objective standards. Second, only when changes distinguishable for human eyes appear can the features be detected and measured, and a number of early subtle changes have consequently been neglected. Thirdly, these clinical features which only described the morphology of optic disc are surely not enough to cover various changes of color, texture and so on. In that case, radiomic analysis is a powerful tool to elucidate subtle relationships between image characteristics and disease status through automated high-throughput feature extraction.

Recently, ophthalmological image analysis based on radiomics has achieved preliminary success in the prediction of disease prognosis and treatment effectiveness. A hybrid prediction model, composed of radiomics imaging features of OCT, demographic and visual factors in non-exudative age-related macular degeneration eyes, provided risk scores of exudation in 3 months with an area under the receiver operating characteristic curve (AUC) of 0.82 (33). For the prediction of drug effectiveness, Feng et al. (34), derived radiomic features from OCT images of patients with choroidal neovascularization and cystoid macular edema before giving anti-vascular endothelial growth factor treatment, based on which the model achieved automatic prediction of treatment effectiveness with an AUC of 0.80. In addition, a small-scale clinical trial identified two radiomics biomarkers from ultra-widefield fluorescein angiography imaging for predicting treatment durability of longer treatment intervals in diabetic macular edema with an AUC of 0.77 (35). Studies above manifested that, as a combination of medical imaging with engineering, radiomics has great potential to be a novel and powerful tool of future precision medicine in the field of ophthalmology.

Although the utility of imaging features has been frequently discussed in machine learning (36), fundus changes of myopia still lack sufficient attention. Recently, Medeiros et al. (37), constructed a model to predict RNFLT from fundus photograph as a risk indicator of glaucomatous damage. Furthermore, a retrospective cohort study validated that longitudinal changes of RNFLT predicted based on fundus photographs forecasted future conversion of glaucomatous visual field defects (38). Color fundus photograph has advantages of low cost and easy access while provides abundant information of morphology, color and texture, while imaging features by radiomics and machine learning provides an effective way to quantify these imaging characteristics. Combining these two methods makes it possible to analyze whether changes of the optic disc and peripapillary regions are related to choroidal thinning and the progression of pathological myopia with statistical methods.

In the present study, by using methods of machine learning, the imaging features of optic disc and peripapillary region were screened for features most correlated with choroidal thickness, which formed new models of risk indicators for changes of choroidal thickness. The new indicators constituted of several imaging features have several advantages. First, they were subjective and quantifiable indexes that required no manual measurements but standardized algorithms to automatically extract features from fundus photographs. Second, whether the changes were visible to the human eye, quantifiable results could be automatically acquired. Third, imaging features by machine learning covered information of color and texture in addition to morphology. All these advantages offset the shortcomings of previous clinical features.

This study had several limitations. First, although the fundus photograph provides abundant information related to morphology, color and texture of optic disc and peripapillary region, only features related to morphology and color of peripapillary region were selected by LASSO regression and enrolled in the final models. One possible reason is that indicators of other information need further designing and mining. However, it is gratifying that models constructed with only selected indicators have significantly stronger correlation than validated indicators including PPA area, ovality index and torsion angle. Second, the age of participants in the present study is limited between 16 and 40 years old, so the results may not be representative of all myopia populations. Finally, the cross-sectional study was not able to determine the causal relationships between changes of quantifiable models and ChT. Whether the changes of optic disc and peripapillary region leads to the progression of choroid thinning or pathological myopic needs further longitudinal studies.

In conclusion, the cohort study explored the possibility of finding non-invasive imaging-based risk indicators indistinguishable for human eyes from fundus photographs with the goal of predicting changes of choroidal thickness and the progression of pathological myopia from early changes of the optic disc and peripapillary region. New models of risk indicators by machine learning provided new ideas for exploring the early changes of optic disc in myopia and its impact on choroidal thickness, which could be useful in other areas of fundus diseases.

## Data Availability Statement

The original contributions presented in the study are included in the article/supplementary material, further inquiries can be directed to the corresponding author/s.

## Ethics Statement

The studies involving human participants were reviewed and approved by the ethics committee of Shanghai General Hospital. Written informed consent to participate in this study was provided by the participants' legal guardian/next of kin.

## Author Contributions

DS, YF, and XX designed this study. DS, YD, HC, and ML collected and measured data. DS, QC, LY, and JH analyzed data. DS, QC, and YF wrote the article. All authors discussed the results and commented on the manuscript.

## Conflict of Interest

The authors declare that the research was conducted in the absence of any commercial or financial relationships that could be construed as a potential conflict of interest.
